# Supplementary material for: Bevacizumab-Based Chemotherapy Triggers Immunological Effects in Responding Multi-Treated Recurrent Ovarian Cancer Patients by Favoring the Recruitment of Effector T Cell Subsets
Source: J Clin Med. 2019 Mar 18;8(3):380. doi: 10.3390/jcm8030380 (PMC6462947; doi:10.3390/jcm8030380)
Supplement: Supplementary file 1 [file jcm-08-00380-s001.pdf]

Table S1. Ca125 levels of patients belonging to Bev- and Ctrl-group

Bev-group			Ctrl-group		
**Responders**			**Responders**		
**T0**	**III**	**VI**	**T0**	**III**	**VI**
1109	749	650	315	201	194
41.2	10.5	7.3	54	18	8
166	38.8	27.3	25	21	25
274	48.7	13.4	25	25	28
2035	291	293	9947	7385	4431
**Not-Responders**			**Not-Responders**		
**T0**	**III**	**VI**	**T0**	**III**	**VI**
98.8	46.1	254.7	12.4	9.06	12
176	186	344	82	95.6	98.3
310	192	1028	9	12	11
714	1630	1522	230	322	530
177	6104	6023	12	11	15
